# Imsnc761 and DDX6 synergistically suppress cell proliferation and promote apoptosis via p53 in testicular embryonal carcinoma cells

**DOI:** 10.1042/BSR20180271

**Published:** 2018-07-03

**Authors:** Zhengzheng Duan, Ping Ping, Guishuan Wang, Xiansheng Zhang, Fei Sun

**Affiliations:** 1 Department of Cell and Developmental Biology, School of Life Sciences, University of Science and Technology of China, Hefei, Anhui 230027, China; 2 Center for Reproductive Medicine, Renji Hospital, School of Medicine, Shanghai Jiao Tong University, Shanghai 200135,China; 3 Medical School, Institute of Reproductive Medicine, Nantong University, Nantong, Jiangsu 226001, China; 4 Department of Urology, The First Affiliated Hospital of Anhui Medical University, Hefei, Anhui 230032, China; 5 Hefei National Laboratory for Physical Sciences at Microscale and School of Life Sciences, University of Science and Technology of China, Hefei, Anhui 230026, China

**Keywords:** apoptosis, DDX6, imsnc761, KEGG pathways, proliferation, p53

## Abstract

Intermediate-sized non-coding RNAs (imsncRNAs) have been shown to play important regulatory roles in the development of several eukaryotic organisms. In the present research, we selected imsncRNA 761 (imsnc761) as a research target. Expression analyses in a previous study showed that imsnc761 was down-regulated in maturation-arrested testis tissues as compared with the level in normal controls. In the present study, we found that imsnc761 could interact with DEAD-box helicase 6 (DDX6) to induce NTERA-2 (NT2 (testicular embryonal carcinoma cell)) cell apoptosis and proliferation inhibition via the p53 pathway. This interaction between imsnc761 and DDX6 also inhibited mitochondrial function and specific gene transcription and translation. To facilitate further research, we used label-free quantitation method to analyze the associated differences in Kyoto Encyclopaedia of Genes and Genomes (KEGG) pathways and biological processes. This confirmed the changes in several specific pathways, which matched our molecular experimental results.

## Introduction

Large-scale studies have clarified that only 2% of all human genome transcripts are translated into proteins [1,2]. The vast majority of transcripts that do not encode proteins, were initially considered ‘junk’ or experimental artefacts, but substantial evidence has now shown that these RNA molecules have biological functions [2,3]. Indeed, the significance of non-coding RNAs (ncRNAs) in mammalian biology has been demonstrated by many studies. Generally, ncRNAs of less than 200 nts in length are classified as short, and those longer than 200 nts are considered as long ncRNAs (lncRNAs). There are also several subtypes of ncRNA and short ncRNA species, most of which are involved in regulation of gene expression and epigenetics [3–5].

Intermediate-sized ncRNAs (imsncRNAs), with length ranging from 50 to 500 nts, have also recently attracted attention. The limited published data on these molecules suggest that imsncRNAs play specific regulatory roles in the development of several eukaryotic organisms [6–8]. However, they have barely been explored in humans. To discover novel human ncRNAs, we used a previously described strategy to construct an ncRNA-specific full length library from human testes. ImsncRNAs (50–500 nts) were extracted from human testis tissue, cloned, and sequenced. Using Illumina/Solexa paired-end sequencing with an imsncRNA-specific library, we performed a systematic identification of novel imsncRNAs in humans, including antisense, intergenic, and intronic imsncRNAs. Based on unpublished sequencing results, we selected imsncRNA 761 (imsnc761) as a research target, which is up-regulated in the hypospermatogenesis patients. Imsnc761 is 129 nts long, and its transcription direction is consistent with *TPT1* mRNA. Recently, it is also identified as a small nucleolar RNA, H/ACA box 31B (SNORA31B) [9].

DEAD-box helicase 6 (DDX6) is a member of the ATP-dependent DEAD-box RNA helicase family and is functionally and evolutionarily conserved amongst eukaryotes [10,11]. Functional studies have demonstrated that DDX6 proteins play critical roles in several biological progresses, such as mRNP assembly and export, RNA degradation, and translational regulation [12–14]. Thus, the DDX6 homolog ste13 in yeast is indispensable for sexual reproduction [15]. The *Xenopus laevis* and *Drosophila melanogaster* homologs, Xp54 and Me13B, respectively, are integral components of stored mRNPs in oocytes [16,17]. Moreover, DDX6 has been shown to play important roles in gametogenesis and early embryogenesis in mice [18,19]. However, the function of DDX6 in the human reproductive system is still undetermined.

Here, we characterized the expression of imsnc761 in the human testes tissues and demonstrated that imsnc761 and DDX6 synergistically inhibited cell proliferation and induced apoptosis in the testicular embryonal carcinoma cell line NTERA-2 (NT2 (testicular embryonal carcinoma cell)). To further investigate the mechanism involved, we used a label-free quantitation method to identify the changed pathways.

## Materials and methods

### Human testicular samples

Human testicular biopsy specimens were obtained from 13 patients with maturation arrest, 6 patients with hypospermatogenesis, and 13 control individuals. Testicular cancer specimens were obtained from four patients and prostate cancer specimens were from three patients. All specimens were obtained from the First Affiliated Hospital of Anhui Medical University (Hefei, China). Testicular biopsy samples were obtained from patients who were undergoing orchiectomy for prostate carcinoma before chemotherapy and who had a history of normal spermatogenesis and fertility and demonstrated normal spermatogenesis. All the patients signed the informed consent documents approving the use of their tissues for research purposes. Written informed consent, which conformed to the tenets of the Declaration of Helsinki, was obtained from each participant prior to the study. The present study received ethical approval from the Institutional Review Boards of the University of Science and Technology of China and Anhui Medical University. All the methods strictly abided by the Ethical Review Organizations’ Guidelines.

### Vectors

The pcDNA3.1 vector and the PEGFP-C1 vector were kindly donated by Mian Wu (University of Science and Technology of China). The DDX6 expression vector was constructed by cloning human DDX6 cDNA into the p3XFLAG-myc-CMV™-24 expression vector and the PEGFP-C1 expression vector. For pcDNA3.1-imsnc761 (imsnc761), imsnc761 was inserted into the pcDNA3.1 vector. For construction of the expression plasmids, total RNA isolated from NT2 cells and human testicular tissues was reverse-transcribed to cDNA. The full-length cDNA was amplified by PCR using RT-PCR primers.

All of the generated constructs were verified by sequencing. The RT-PCR primer sequences are listed in Table 1.

**Table 1:** Sequences and primers

**Oligonucleotides**	**Sequence (5′–3′)**	
imsnc761	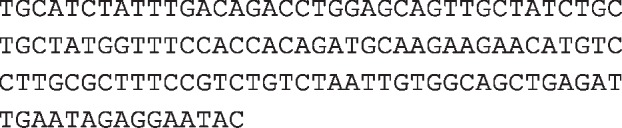	
imsnc761 antisense	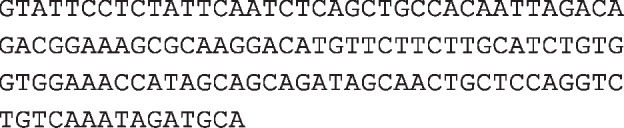	
LNA imsnc761	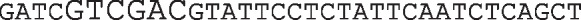	
**Gene**	**Forward primer sequence (5′–3′)**	**Reverse primer sequence (5′–3′)**
*PEGFP-C1-DDX6*	ATTAGATCTATGAGCACGGCCAGAACAGAG	TGCGTCGACTTAAGGTTTCTCATCTTCTACA
*p3XFLAG-DDX6*	ATTGCGGCCGCATGAGCACGGCCAGAACAGAG	TGCGTCGACTTAAGGTTTCTCATCTTCTACA
*pcDNA3.1(+)-imsnc761*	CGCGGATCCTGCATCTATTTGACAGACCTGG	CCGGAATTCGTATTCCTCTATTCAATCTCAGCT
*imsnc761 real-time PCR*	GACCTGGAGCAGTTGCTATC	TGCCACAATTAGACAGACGGAAAG
*β-actin real-time PCR*	TGGCACCCAGCACAATGAA	CTAAGTCATAGTCCGCCTAGAAGCA

Abbreviations: LNA, locked nucleic acid.

### RNA extraction and real-time PCR

Total RNA was isolated with TRIzol (Invitrogen, CA, U.S.A.) from NT2 cells and human testicular tissues and then reverse-transcribed into cDNA using a PrimeScript RT reagent kit (TaKaRa Bio Inc., Otsu, Japan). Real-time PCR was performed on an ABI Step One System (Applied Biosystems, Foster City, CA, U.S.A.) thermocycler using the SYBR Premix Ex Taq kit (Takara Bio Inc.), in accordance with the manufacturers’ protocols. mRNA expression levels were normalized to human *GAPDH* mRNA expression. The real-time PCR primer sequences are listed in Table 1.

### Cell culture and transfection

NT2 and HEK293T cells were cultured in Dulbecco’s modified Eagle’s medium (DMEM) supplemented with 10% (v/v) FBS (Life Technologies Inc., CA, U.S.A.) and 1% antibiotics (100 units/ml penicillin and 100 μg/ml streptomycin, Life Technologies Inc., Grand Island, NY, U.S.A.). The cells were cultured at 37°C in a 5% carbon dioxide atmosphere. We used Lipofectamine RNAiMAX (Invitrogen, Carlsbad, CA, U.S.A.) and X-tremeGENE HP DNA Transfection Reagent (Roche, Basel, Switzerland) to transfect the NT2 cells with oligonucleotides and plasmids. The Lipofectamine 3000 Reagent (Invitrogen, CA, U.S.A.) was used to transfect HEK293T cells. All transfection procedures were performed following the manufacturers’ instructions.

### *In situ* hybridization

The expression of imsnc761 in the human testicular biopsy specimens was examined by *in situ* hybridization (ISH) using locked nucleic acid (LNA)-modified DNA probes on 8-μm frozen tissue sections as described by Lian et al. [20]. Briefly, 8-μm testis biopsy sections were prehybridized for 6 h at 55°C with 700 μl prehybridization buffer (50% formamide, 5× saline sodium citrate, 5× Denhardt’s, 200 μg/ml yeast RNA, 500 μg/ml salmon sperm DNA, 2% Roche blocking reagents (Roche, Basel, Switzerland), and DEPC-treated water). Sections were then covered with 150 μl denatured hybridization buffer containing 1 pmol LNA probe and incubated overnight at 52°C. The hybridization signals were examined with anti-digoxigenin (DIG)-alkaline phosphatase fragments from antigen binding fragments (1:250; Roche) and nitro blue tetrazolium/5-bromo-4-chloro-3-indolyl phosphate solution (NBT/BCIP) (Roche).

### Immunoprecipitation assays

Native RNA immunoprecipitation (RIP) was performed with the EZ-Magna RIP Kit (Millipore) in accordance with the manufacturer’s protocol using 20 μg rabbit anti-GFP antibody or rabbit IgG. The co-precipitated RNAs were extracted using TRIzol (Invitrogen) and detected by the real-time PCR. The proteins that were isolated from the beads were detected by immunoblotting.

### RNA pull-down assay

The imsnc761 cDNA was cloned into the pSPT18/19 vector. Biotin-labeled RNAs were transcribed *in vitro* by SP6 RNA polymerase (Roche) and purified using the MEGAscript Kit (Ambion, CA, U.S.A.). Cells were harvested and resuspended in freshly prepared binding buffer (10 mM HEPES pH 7.0, 50 mM KCl, 10% glycerol, 1 mM EDTA, 1 mM DTT, and 0.5% Triton X-100) supplemented with tRNA (0.1 μg/μl), heparin (0.5 μg/μl), and RNasin (1 U). Biotin-labeled RNAs (3 μg) were mixed with 200 μg whole-cell lysate and then mixed with pre washed streptavidin-coupled Dynabeads (Invitrogen) for 1 h at RT. The beads were then washed with ice-cold binding buffer for five times, after which they were boiled in 2× Laemmli loading buffer. The retrieved proteins were subjected to SDS/PAGE and further visualized by silver staining or immunoblotting. Protein bands were excised and identified by in-gel trypsin digestion followed by MS analysis.

### Histological analysis and immunohistochemistry

Immunohistochemistry (IHC) was conducted to localize the DDX6 protein in human testicular tissues. Human testes were dissected into pieces, fixed with 4% PFA, embedded in paraffin, and sectioned at 4 μm. To confirm the specific infertility syndrome, sections were stained with Hematoxylin and Eosin following a standard protocol. Initially, slides with testicular tissue sections were heated in 10 mM sodium citrate buffer (pH 6.0) for 10 min, after deparaffinization in a microwave oven. The sections were then immersed in PBS containing 3% H_2_O_2_ and 0.1% Triton X-100 to quench endogenous peroxidase activity. After treatment with 10% normal donkey serum (Jackson ImmunoResearch Labs Inc., West Grove, PA, U.S.A.) to block non-specific binding signals, the slides were incubated with DDX6-specific antibody (GeneTex, CA, U.S.A.) overnight at 4°C and then incubated with a mouse biotinylated secondary antibody (Abcam, Cambridge, MA, U.S.A.) for 2 h at room temperature. Immunoreactivity with DDX6 was visualized using streptavidin-peroxidase and 3,3′-diaminobenzidine (Maixin Bio, Fuzhou, China).

### Cell proliferation assay

CCK-8 (Dojindo Laboratories, Kumamoto, Japan) was used to measure cell proliferation. NT2 cells were plated at 3–5×10^3^ cells per well in 96-well plates with five replicate wells for each condition. Forty-eight hours after transfection, the culture medium from each well was treated with 10 μl CCK-8 solution and then incubated for 2 h at 37°C. The cell numbers were measured by measuring the absorbance at 450 nm (OD450) using a 96-well format plate reader (ELx800 Universal Microplate Reader; BioTek Instruments Inc., Highland Park, VT, U.S.A.).

### Flow cytometry

Apoptosis was detected using a Cytomics™ FC 500 flow cytometer (Beckman Coulter, CA, U.S.A.). For the analysis of apoptosis, the cells were seeded in six-well plates at 30–50% confluence. The cells were harvested at 48 h after transfection with pcDNA3.1-imsn761 and p3XFLAG-DDX6 stained with an Annexin V FITC Apoptosis Detection Kit (Vazyme Biotech Co., Ltd., Nanjing, China), and analyzed by flow cytometry.

### Determination of ATP function

The ATP function of cells was determined using a Luminescent ATP Detection Assay Kit (Abcam). The cells were harvested at 48 h after transfection with pcDNA3.1-imsn761 and p3XFLAG-DDX6. After treatment, cells were lysed, exposed to ATP substrate solution, and the signal was measured on a luminescent counter. Mean and S.D. were plotted for three replicates from each condition.

### Western blotting

Cells were lysed in RIPA buffer (50 mM Tris/HCl pH 7.4, 150 mM NaCl, 1% Triton X-100, 1% SDS, 1% sodium deoxycholate, and 1 mM EDTA) containing complete EDTA-free protease inhibitor cocktail (Roche), 1 mM PMSF, and phosphatase inhibitors (5 mM sodium orthovanadate). Protein lysates were separated by SDS/PAGE, electrically transferred to a Hybond ECL nitrocellulose membrane (Amersham Biosciences, Freiburg, Germany), immunoblotted with antibodies and visualized by ECL (Kodak, Rochester, NY, U.S.A.). Protein levels were normalized to the level of β-actin. The following primary antibodies were used for immunoblotting: anti-Actin (Abcam, Cambridge, MA, U.S.A.), anti-MAPK, anti-IKK-β, anti-PARP, anti-caspase9 (Cell Signaling Technology, MA, U.S.A.), anti-CCND1, anti-CDK4 (Santa Cruz Biotechnology, CA, U.S.A.).

### ncRNA library construction

Total RNA was isolated from the human testis tissues according to the TRIzol (Invitrogen) protocol. One milligram total RNA from four different patients was pooled and mixed in equal aliquots, then the total RNA mix was loaded on a Qiagen RNA/DNA maxi column (Qiagen). The following procedures were performed essentially as described previously [8,21].

### Northern blotting

Northern blots were carried out according to the manufacturer’s protocol (DIG Northern Starter Kit, Roche). DIG-labeled imsnc761 and U6 probes were made using either SP6 or T7 RNA polymerases by *in vitro* transcription with the DIG Northern Starter Kit (Roche) [22].

## Results

### The sequence information and identification of imsnc761

**Figure 1:**
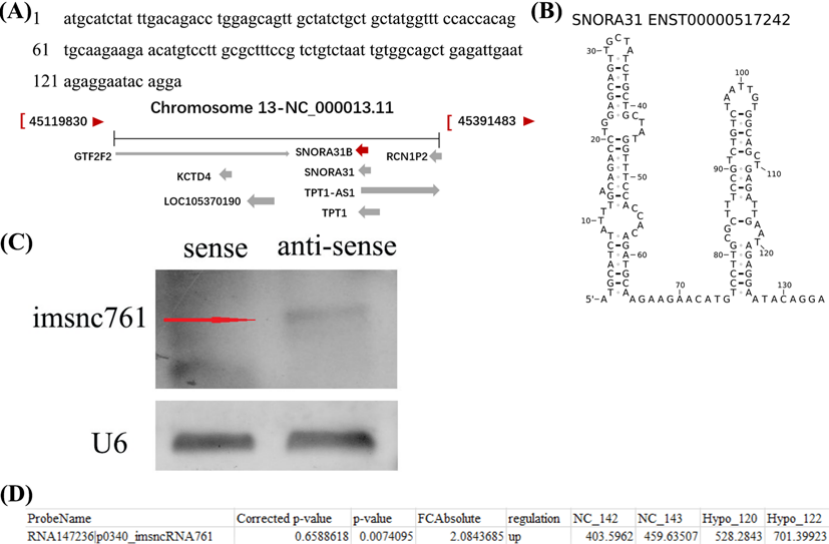
The sequence information and identification of imsnc761 (**A**) The sequence and location of imsnc761. (**B**) The secondary structure of imsnc761 in the database e!Ensembl (www.ensembl.org). (**C**) The identification of imsnc761 by northern blotting. (**D**) The data provided in the table represent the imsnc761 that were found to be up-regulated significantly in hypospermatogenesis patients compared with controls.

Imsnc761 was identified as an imsncRNA by using a specific ncRNA library first. But recently the sequence of imsnc761 was listed as a snoRNA, which named *Homo sapiens* SNORA31B. Although the way we named was different, but the sequence was completely matched (Figure 1A). Even the NCBI database showed the information of the sequence and related publication, the role of the imsnc761/SNORA31B was still unknown. Imsnc761 was located in 13q14.13 (Figure 1A). As we already know, H/ACA box snoRNAs have a common secondary structure consisting of two hairpins and two single-stranded regions termed as hairpin-hinge-hairpin-tail structure. H/ACA snoRNAs also contain conserved sequence motifs known as H box (consensus ANANNA) and the ACA box (ACA). From the database e!Ensembl (www.ensembl.org), imsnc761/SNORA31B (Ensembl version ENSG00000253051.1) contains a typical snoRNA secondary strcuture (Figure 1B).

According to that information, northern blotting was applied to verify the existence and expression of imsnc761. The DIG-labeled imsn761 sense sequence and imsn761 antisense sequence were used as probe. The results showed that imsnc761 existed in NT2 cells (Figure 1C). Based on our sequencing results, a clear change of imsn761 was displayed between normal controls and hypospermatogenesis patients (Figure 1D).

### Imsnc761 localized in spermatogonia and spermatocytes, was down-regulated in testicular cancer

**Figure 2:**
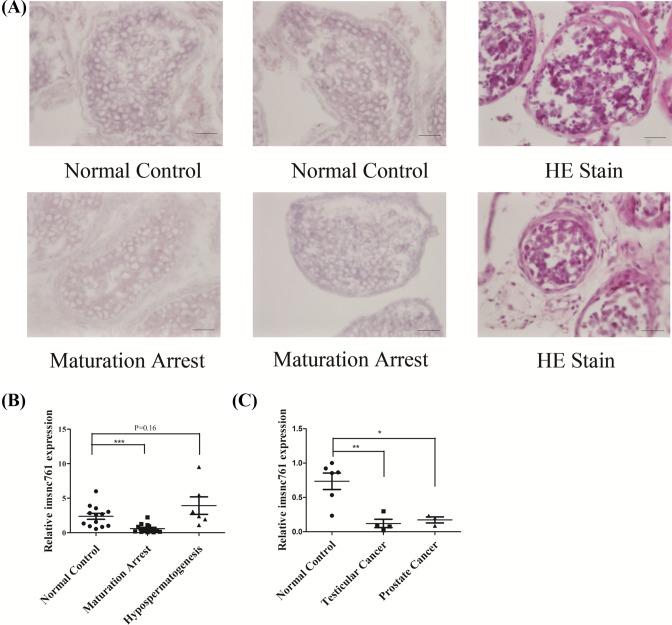
Imsnc761 localized in spermatogonia and spermatocytes, was down-regulated in testicular cancer (**A**) Localization of imsnc761 in the testes of normal controls and maturation arrest patients by LNA-based miRNA ISH. (**B**) The expression of imsnc761 in the testes of normal controls and patients with maturation arrest and hypospermatogenesis was examined by real-time PCR. (**C**) The expression of imsnc761 in the testes of normal controls and patients with testicular cancer and prostate cancer was examined by real-time PCR. Scale bar = 50 µm. All data are presented as the means ± S.E.M. from at least three independent experiments. *, *P*<0.05; **, *P*<0.01; ***, *P*<0.001.

The expression of imsnc761 in testicular biopsy specimens was examined by ISH. In seminiferous tubules with normal spermatogenesis, imsnc761 expression was predominantly found in the spermatogonia and primary spermatocytes. The expression of imsnc761 in patients with maturation arrest was consistent with that in normal controls (Figure 2A).

In addition, imsnc761 was significantly decreased in specimens with maturation arrest as determined by real-time PCR. This down-regulation may not be exclusive to these patients, as imsnc761 was highly expressed in infertile patients with hypospermatogenesis (Figure 2B). These results suggested that imsnc761 may play a role in non-obstructive azoospermia. Besides, imsnc761 was down-regulated in testicular cancer and prostate cancer specimens (Figure 2C). These findings indicate that imsnc761 is associated with testicular and prostate cancer.

### Imsnc761 interacted with DDX6

**Figure 3:**
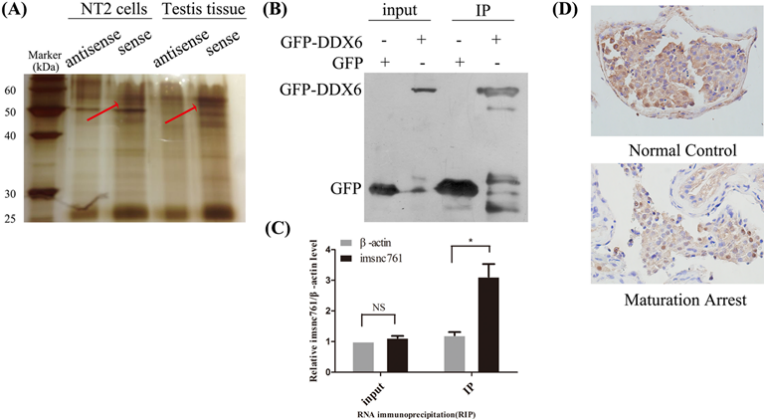
Imsnc761 interacted with DDX6 (**A**) Identification of cellular proteins associated with imsnc761. Proteins from NT2 cell and human testes tissues extracts were pulled down with the biotinylated RNAs, subjected to SDS/PAGE, and visualized by silver staining. The bands specific to imsnc761, as indicated by the red arrow, were subjected to MS. (**B**,**C**) Imsnc761 interacts with DDX6 *in vivo*. DDX6 was IP from NT2 cells and co-precipitated RNAs were detected by qRT-PCR using primers for imsnc761 and β-actin (as negative control). IP enrichment was determined as the amount of RNA associated with the DDX6 IP relative to IgG control. (**D**) Immunohistochemical analysis of the DDX6 protein in human testicular tissue from patients with maturation arrest. Scale bar = 50 μm for all images. Data are from one of three independent experiments and are represented as means ± S.E.M. from at least three independent experiments. *, *P*<0.05.

As previous studies have shown that ncRNAs could function by interacting with proteins, we assumed that the role of imsnc761 in biological processes might be mediated by a similar mechanism. To identify the proteins that are associated with imsnc761, we performed pull-down assays with biotinylated imsnc761. Compared with antisense imsnc761 (lanes 2 and 4 from the left), there were different bands that specifically associated with imsnc761 (lanes 3 and 5 from the left). We focussed on the bands that only occurred in samples with imsnc761 sense sequences. The proteins specific to imsnc761 were extracted, digested with trypsin, and subjected to MS analysis (Figure 3A). In the group using NT2 cells, approximately 34 proteins were identified. Meanwhile, in the group using testis tissue, 50 proteins were identified. Several proteins overlapped between the two groups, such as cDNA FLJ51294, citrate synthase, and DDX6. These proteins were chosen for further analysis. DDX6 was shown to interact with imsnc761. The association between imsnc761 and DDX6 was validated by RIP. Imsnc761 was clearly detected amongst the RNAs associated with DDX6 but was not found in the complexes associated with the IgG control *in vivo* (Figure 3B,C). Immunohistochemical analyses showed that DDX6 was localized in the spermatogonia and primary spermatocytes in the testes of normal control and patients with maturation arrest (Figure 3D), consistent with the localization of imsnc761 in human testicular tissues.

### Imsnc761 and DDX6 synergistically inhibited NT2 cell growth and promoted apoptosis via p53

**Figure 4:**
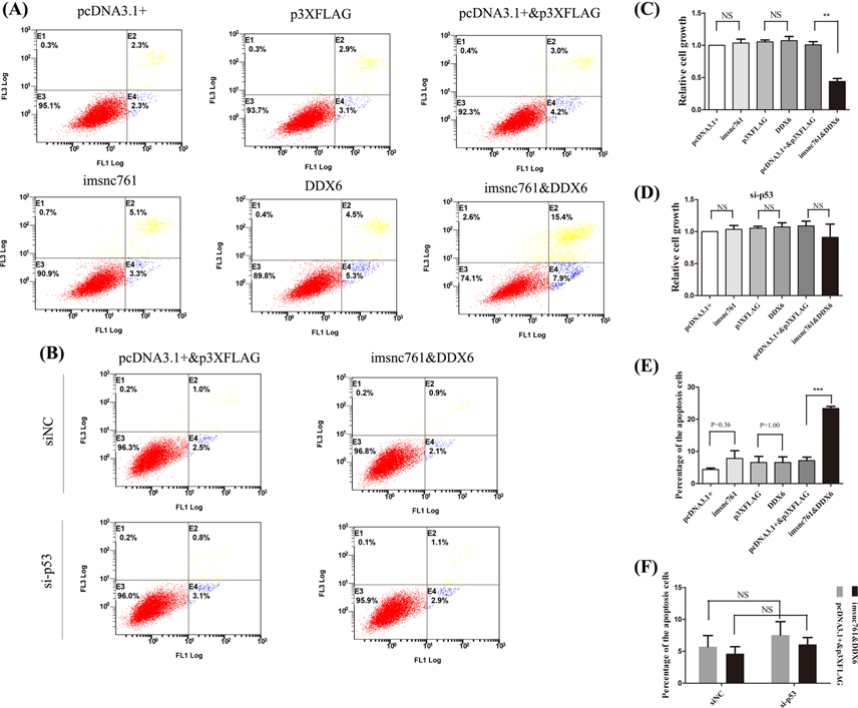
Imsnc761 and DDX6 synergistically inhibited NT2 cell growth and promoted apoptosis via p53 (**A**) Co-expression of imsnc761 and DDX6 induced cell apoptosis. The apoptosis rate was measured using flow cytometry with Annexin V and PI staining after transfection either with 2 μg plasmids. (**E**) The percentage of the apoptosis cells of the statistical results. (**C**) Co-expression of imsnc761 and DDX6 inhibited cell proliferation. Cell viability was measured by the CCK-8 assay after transfection either with 2 μg plasmids. (**D**) The inhibition of cell growth induced by imsn761 and DDX6 rescued by si-p53. (**B**) The apoptotic effect induced by imsnc761 and DDX6 rescued by si-p53. (**F**) The apoptotic effect induced by imsnc761 and DDX6 rescued by si-p53. All data are presented as the means ± S.E.M. from at least three independent experiments. **, *P*<0.01; ***, *P*<0.001; NS, not significant

To evaluate the effects of imsnc761 on NT2 cell survival, we transfected a construct for overexpressing imsnc761 into NT2 cells. The results showed that imsnc761 did not affect NT2 cell proliferation (Figure 4C). FACS analysis was also performed to confirm that imsnc761 did not promote NT2 cell apoptosis (Figure 4A,E). To investigate the synergistic effects of imsnc761 and DDX6, overexpressed endogenous imsnc761 and DDX6 were transfected into NT2 cells. The CCK8 assay showed that the proliferation of cells co-transfected with imsnc761 and DDX6 was significantly reduced compared with that in the control groups (Figure 4C). Meanwhile, flow cytometry demonstrated that imsnc761 and DDX6 co-overexpression significantly increased apoptosis compared with that in the control groups (Figure 4A,E).

p53-induced apoptosis is a critical mechanism for suppressing tumor development [23–26]. As such, to investigate whether overexpression of imsnc761 and DDX6 could induce apoptosis in the absence of p53, imsnc761/pcDNA3.1+, DDX6/p3XFLAG, and si.P53/si.NC were co-transfected into NT2 cells, and 48 h later apoptosis was detected by FACS. The results showed that in the absence of p53, the overexpression of imsnc761 and DDX6 could not induce any apoptosis of NT2 cells (Figure 4B,F). Meanwhile, we found that the inhibition of cell proliferation induced by imsn761 and DDX6 were also dependent on p53 (Figure 4D).

### Imsnc761 and DDX6 synergistically inhibited mitochondrial function and the transcription or translation of certain genes

**Figure 5:**
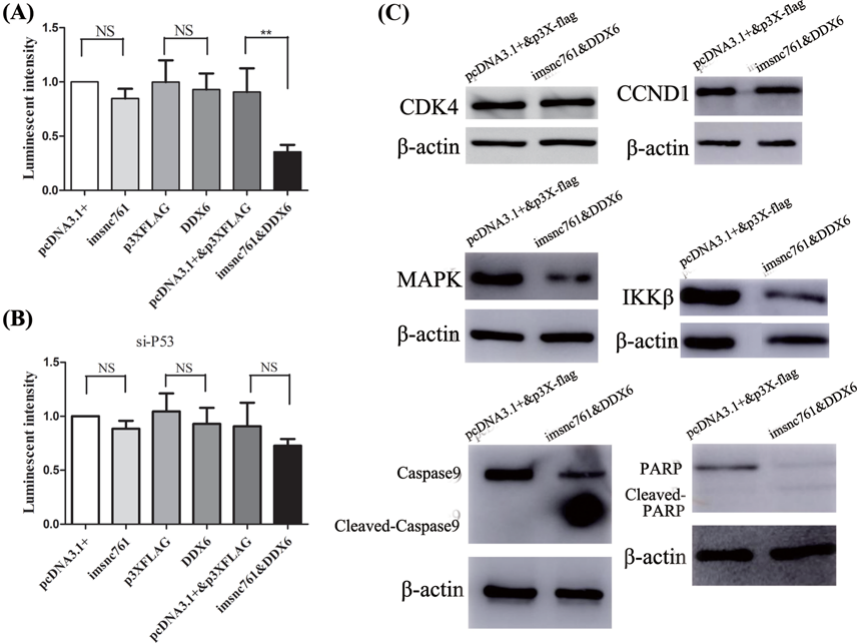
Imsnc761 and DDX6 synergistically inhibited mitochondrial function and the transcription or translation of certain genes (**A**) The ATP luminescent intensity decreased by co-expression of imsnc761 and DDX6. The luminescent intensity was measured by Luminescent ATP Detection Assay Kit from Abcam. After transfection for 48 h, cells were lysed, exposed to the ATP substrate solution and signal was measured on a luminescent counter. (**B**) The down-regulation of luminescent induced by imsnc761 and DDX6 rescued by si-p53. (**C**) The expression of related genes in protein level. All data are presented as the means ± S.E.M. from at least three independent experiments. **, *P*<0.01; NS, not significant.

Since apoptosis is related to mitochondrial function and ATP [27–29], we determined the effects of imsnc761 and DDX6 on mitochondrial function using a Luminescent ATP Detection Assay Kit. The results showed that mitochondrial function (as represented by ATP activity) was impaired in the cells co-expressing imsnc761 and DDX6, but that the other groups showed no difference in this regard (Figure 5A). Moreover, when we co-expressed si-p53, imsnc761/pcDNA3.1(+), and DDX6/p3XFLAG into NT2 cells, the amount of ATP returned to the normal level (Figure 5B).

Since the apoptotic effect was significant, we wondered which genes exhibited a change of expression in association with this. To investigate this, we used Western blotting to examine certain genes related to the apoptosis signaling pathway. The result showed that the expression of IKK-β and MAPK was significantly decreased. Meanwhile, the expression of genes related to the cell cycle, such as *CCND1* and *CDK4* did not change. A member of the caspase family, caspase 9 and its downstream gene *PARP* were activated and cleaved (Figure 5C).

### Proteomics in NT2 cells transfected with imsnc761 and DDX6

**Figure 6:**
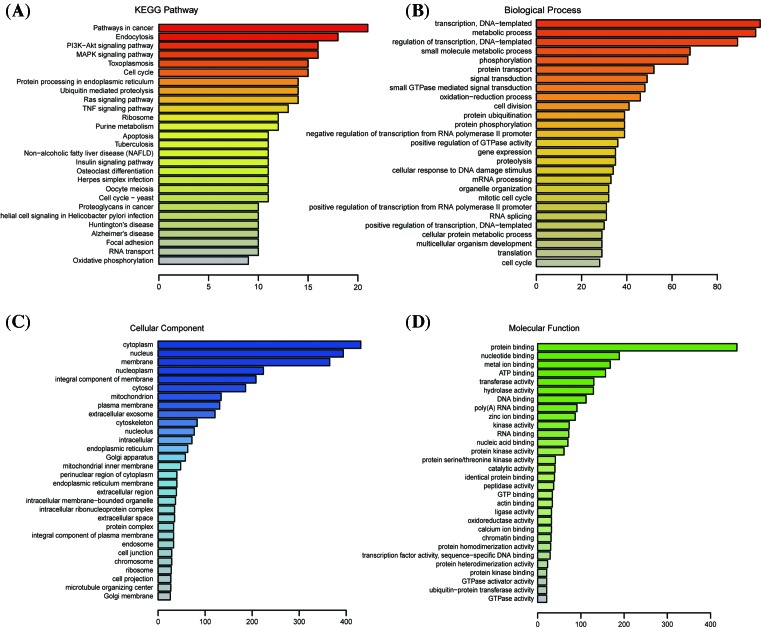
Proteomics in NT2 cells transfected with imsnc761 and DDX6 (**A**) Differentially expressed proteins were classified by KEGG pathway enrichment analysis. (**B**–**D**) GO on biological process, cellular component, and molecular function.

Substantial progress has recently been made in the field of quantitative proteomics [30,31]. Label-free quantitative proteomics analysis based on the MS had been widely used [32–34]. To study the underlying mechanisms, we conducted a functional proteomics assay. The experiment featured two separated groups, namely NT2 cells transfected with imsnc761 and DDX6 and NT2 cells transfected with the corresponding concentration of the control vector; each experiment was performed in triplicate. Kyoto Encyclopaedia of Genes and Genomes (KEGG) pathway enrichment analysis was performed to explore the functions of imsnc761 + DDX6. According to the KEGG results, the differentially expressed proteins could be classified into 27 groups. The most abundant proteins were involved in pathways in cancer and endocytosis. Some of the pathways with which we were concerned have also undergone significant changes, such as the MAPK signaling pathway, cell cycle, and apoptosis (Figure 6A). Besides, according to the KEGG analysis, there were several genes’ expression had changed which were related to p53 pathway: STEAP3, Caspase 3, CDKN2A, ATR, EI24, PIRH2. STEAP3-mediated downstream responsed to p53, including promoting apoptosis [35,36]. PIRH2-mediated E3-dependent ubiquitination and proteasomal degradation of target proteins, including tumor protein 53, histone deacetylase 1, and cyclin-dependent kinase inhibitor 1B, thus regulating their levels and cell cycle progression [37–39]. We also performed Gene Ontology (GO) analysis to survey the biological processes (Figure 6B). The categories of transcription and metabolic were significantly changed, including regulation of transcription and small molecular metabolic process. At the same time, analysis of cellular components showed that changes were focussed on the cytoplasm and nucleus (Figure 6C). Protein binding and nucleotide binding had significant differences in molecular function (Figure 6D).

## Discussion

Recently, many studies have shown that transcription of the human genomes is not only widespread but also enormously complicated. The finding that the proportion of non-protein coding DNA is as high as 98.5% in humans implied that ncRNAs play significant roles in developmental complexity [1,2]. Over the last few decades, numerous ncRNAs have been identified to play considerable roles in the regulating of gene expression at the transcriptional, post-transcriptional, or translational levels. The existence of imsncRNAs has also been demonstrated but they are yet to be deeply explored. This type of ncRNA can play a variety of roles in an organism, including participating in transcription and translation or interacting with other biological elements to regulate biological functions [40–43]. Based on sequencing results (unpublished), we selected to explore the biological function and significance of imsnc761, which is differently expressed in the testes. A series of experiments confirmed that imsnc761 does not significantly affect NT2 cell function, but we were surprised to find out that imsnc761 can interact with the protein DDX6. DDX6 encodes an RNA helicase that is a member of the DEAD-box protein family. The proteins are RNA helicases which were found in P-bodies and stress particles. DDX6 plays a role in translation inhibition and mRNA degradation [44–46].

We found that the high endogenous expression of imsnc761 and DDX6 expression significantly inhibited NT2 cell proliferation and increased apoptosis. After a series of experiments, we confirmed that this was associated with the significant inhibition of apoptosis and ATP function. Co-expression of imsnc761 and DDX6 decreased the function of ATP in NT2 cells along with the apoptotic effect. Since p53 has been shown to be extremely important in tumor cells growth and development [23,47,48], we performed several experiments that showed that the silencing of p53 rescued the abovementioned inhibition of cell proliferation and apoptosis. These results confirmed that imsnc761 and DDX6 induce growth inhibition and apoptosis in a manner dependent on p53. Since apoptosis is a complicated progress, numerous genes and pathways are related to it. We decided to detect some related genes’ expression. IKK-β is a protein subunit of IκB kinase, a component of the intracellular cytokine-activated signaling pathway, which is involved in the triggering immune responses. IKK-β activity has been reported to activate the transcription factor NF-κB [49,50]. MAPK is a protein kinase, that is specific to the amino acids, serine and threonine. It regulated cell proliferation, gene expression, differentiation, mitosis, cell survival, and apoptosis [51–55]. Upon the co-expression of imsnc761 and DDX6, IKK-β and MAPK were significantly down-regulated. In contrast, the expression levels of CCND1 and CDK4 which are related to the cell cycle did not change [56]. The aspartic acid specific protease caspase9 is linked to the mitochondrial death pathway [57,58]. During programmed cell death, caspase9 is activated [59]. *PARP* is a downstream gene of caspase-9 involving in DNA repair, genomic stability, and apoptosis [60,61]. Here, caspase9 and PARP were shown to be activated and cleaved by the overexpression of imsnc761 and DDX6. The changes in the expression of these genes were consistent with their reported functions.

To obtain a more comprehensive understanding of the underlying mechanism, we used label-free quantitative proteomics to show that multiple internal signaling pathways, including apoptosis, cell cycle, and MAPK pathways, had changed in cells treated with imsnc761 and DDX6 compared with the status in the controls. The obtained sequencing results were highly consistent with our experimental results. Recently, the sequence of imsnc761 was identified as a snoRNA, named as SNORA31B. The snoRNAs function in association with specific proteins and thus form ribonucleoproteins (snoRNPs). The action of snoRNPs is essential to the removal of introns from pre-mRNA, a critical aspect of post-transcriptional modification of RNA. The RNA helicase DDX6 could regulate miRNA’s expression [46]. But the relationship between snoRNA and DDX6 was still unknown. The snoRNA usually functioned by binding to proteins and DDX6 was related to RNA processing. We guessed imsnc761 and DDX6 may form a snoRNP complex to induce the apoptosis effect.

Although some unresolved issues remained in our study, this preliminary research revealed that the combination of an imsncRNA and a protein could significantly affect tumor cells. Thus, further studies on the involvement of ncRNAs in the reproductive system and tumors are strongly warranted.

## Abbreviations

CCND1: cyclin D-1
CCK-8: cell counting kit 8
CDK4: cyclin dependent kinase 4
DDX6: DEAD-box helicase 6
DEPC: diethyl pyrocarbonate
DIG: digoxigenin
GAPDH: glyceraldehyde-3-phosphate dehydrogenase
IκB: inhibitor of NF-κB
IKK: IκB kinase
imsncRNA: intermediate-sized non-coding RNA
imsnc761: imsncRNA 761
IP: immunoprecipitation
ISH: *in situ* hybridization
KEGG: Kyoto Encyclopaedia of Genes and Genomes
LNA: locked nucleic acid
MAPK: mitogen activated protein kinase
mRNP: messenger ribonucleoprotein
ncRNA: non-coding RNA
NF-κB: nuclear factor kappa B
NT2: testicular embryonal carcinoma cell
PARP: poly ADP ribose polymerase
PFA: paraformaldehyde
RIP: RNA immunoprecipitation
RIPA: radio immunoprecipitation assay
RT: real time
RT-PCR: realtime polymerase chain reaction
SNORA31B: small nucleolar RNA, H/ACA box 31B
TPT1: tumor protein, translationally-controlled 1
